# Attenuation of Acetylcholine Activated Potassium Current (*I_KACh_*) by Simvastatin, Not Pravastatin in Mouse Atrial Cardiomyocyte: Possible Atrial Fibrillation Preventing Effects of Statin

**DOI:** 10.1371/journal.pone.0106570

**Published:** 2014-10-16

**Authors:** Kyoung-Im Cho, Tae-Joon Cha, Su-Jin Lee, In-Kyeung Shim, Yin Hua Zhang, Jung-Ho Heo, Hyun-Su Kim, Sung Joon Kim, Kyoung-Lyoung Kim, Jae-Woo Lee

**Affiliations:** 1 Cardiovascular Research Institute, Department of Internal Medicine, Kosin University College of Medicine, Busan, South Korea; 2 Department of Physiology, Seoul National University College of Medicine, Seoul, South Korea; 3 Department of Molecular Biology, Kosin University College of Medicine, Busan, South Korea; The Ohio State University, United States of America

## Abstract

Statins, 3-hydroxy-3-methyl-glutaryl-CoA reductase inhibitors, are associated with the prevention of atrial fibrillation (AF) by pleiotropic effects. Recent clinical trial studies have demonstrated conflicting results on anti-arrhythmia between lipophilic and hydrophilic statins. However, the underlying mechanisms responsible for anti-arrhythmogenic effects of statins are largely unexplored. In this study, we evaluated the different roles of lipophilic and hydrophilic statins (simvastatin and pravastatin, respectively) in acetylcholine (100 µM)-activated K^+^ current (*I_KACh_*, recorded by nystatin-perforated whole cell patch clamp technique) which are important for AF initiation and maintenance in mouse atrial cardiomyocytes. Our results showed that simvastatin (1–10 µM) inhibited both peak and quasi-steady-state *I_KACh_* in a dose-dependent manner. In contrast, pravastatin (10 µM) had no effect on *I_KACh_*. Supplementation of substrates for the synthesis of cholesterol (mevalonate, geranylgeranyl pyrophosphate or farnesyl pyrophosphate) did not reverse the effect of simvastatin on *I_KACh_*, suggesting a cholesterol-independent effect on *I_KACh_*. Furthermore, supplementation of phosphatidylinositol 4,5-bisphosphate, extracellular perfusion of phospholipase C inhibitor or a protein kinase C (PKC) inhibitor had no effect on the inhibitory activity of simvastatin on *I*_KACh_. Simvastatin also inhibits adenosine activated *I_KACh_*, however, simvastatin does not inhibit *I_KACh_* after activated by intracellular loading of GTP gamma S. Importantly, shortening of the action potential duration by acetylcholine was restored by simvastatin but not by pravastatin. Together, these findings demonstrate that lipophilic statins but not hydrophilic statins attenuate *I_KACh_* in atrial cardiomyocytes *via* a mechanism that is independent of cholesterol synthesis or PKC pathway, but may be via the blockade of acetylcholine binding site. Our results may provide important background information for the use of statins in patients with AF.

## Introduction

Atrial fibrillation (AF) is the most common type of chronic cardiac arrhythmia [1], [2], and the pathophysiology of AF is complex [3]–[5]. Statins have pleiotropic effects which are independent of their cholesterol-lowering effects [5], [6]. Furthermore, it has been shown that statins can modulate the activities of L-type calcium channels and transient outward potassium channels, which are altered by rapid atrial pacing [7]. These properties can partially explain ionic mechanisms of the anti-arrhythmic effect of statins.

However, clinical trials have shown conflicting results regarding the anti-arrhythmic effects of statins [6], [8]–[11]. In particular, the GISSI Heart Failure (GISSI-HF) trial showed that the hydrophilic statin, rosuvastatin, did not affect clinical outcome and exerted little benefit with regard to AF occurrence [10], [11]. In contrast, simvastatin, a lipophilic statin, has been shown to prevent the occurrence of AF in a rapid atrial pacing animal model [12]. According to the Sarr et al. [13], hydrophilic pravastatin exhibited the lowest association with the lipid monolayer, and lipophilic simvastatin showed a strong membrane elution ability, which can be explained by hydrophobicity of statin molecule [14]. These findings suggest that lipophilic and hydrophilic statins may differ with respect to effects on the myocardium as a result of different ion channel binding affinity. For example, simvastatin may reduce susceptibility to ventricular fibrillation mainly by reducing sympathetic hyperinnervation and electrical remodeling induced by hypercholesterolemia [15]. So, we can hypothesize that simvastatin may modulate membrane ion channel more effectively than hydrophilic pravastatin.

Although simultaneous sympathetic and parasympathetic (sympathovagal) activation may facilitate the onset of paroxysmal AF [16], effects of statins on the neurohormonal imbalances are not known yet [17]. A plausible link between sympathovagal and neurohormonal interactions in cardiac myocytes is the acetylcholine-activated K^+^ current (*I_KACh_*). *I_KACh_* is involved in tachycardia-induced electrical remodeling and participates in AF initiation and maintenance. In atrial cardiomyocytes, *I_KACh_* is constitutively active, and atrial tachycardia may further increase its activity. Considering the evidence that statins may suppress AF, we hypothesized that statins influence *I_KACh_* in atrial myocytes, and that the effects may vary with the lipophilicity of the statin. To test this hypothesis, we compared the effects of the lipophilic simvastatin with effects of the hydrophilic pravastatin on *I_KACh_* and acetylcholine-induced action potential duration (APD) in atrial cardiomyocytes.

## Materials and Methods

### Experimental design

Imprinting Control Region mice weighing 20∼30 g were used for animal experiments. The protocols for animal care and use were in accordance with the NIH Guide for the Care and Use of Laboratory Animals and were approved by the Animal Research Committee at Kosin University Gospel Hospital. To isolate mouse atrial myocytes, the hearts were rapidly excised and mounted onto a Langendorff apparatus at 37°C and perfused with a Ca^2+^-free normal Tyrode solution containing collagenase (0.14 mg/ml). The *I_KACh_* current was recorded using a nystatin-perforated whole cell patch-clamp technique following activation by acetylcholine (100 µM for 2 min). After measurement of the baseline *I_KACh_* current, atrial myocytes were perfused with lipophilic statins (simvastatin 10 µM for 10 min), after which the *I_KACh_* current was re-measured. The *I_KACh_* currents were compared with those measured in the presence of a hydrophilic statin (pravastatin 10 µM for 10 min). We also evaluated the underlying mechanism of simvastatin-induced *I_KACh_* inhibition.

### Isolation of single cardiomyocytes

Ten mice in each group were anesthetized with pentobarbital sodium (50 mg/kg, intraperitoneally). Hearts were removed by thoracotomy and quickly mounted onto a modified Langendorff perfusion system. To ensure coronary circulation, hearts were sequentially perfused with four solutions (all at 37°C) as follows: (1) normal Tyrode's solution containing (in mM): NaCl 143, KCl 5.4, CaCl_2_ 1.8, MgCl_2_ 0.5, NaH2PO_4_ 0.33, HEPES 5 and glucose 10, adjusted with NaOH to pH 7.4, for 4–5 min; (2) Ca^2+^-free normal Tyrode's solution for 5 min; (3) Ca^2+^-free normal Tyrode's solution supplemented with collagenase (type II, 15 mg/35 ml, Worthington, USA) for 15–20 min; and (4) a high K^+^, low-Cl^−^ solution (modified Kraft-Brühe [KB] solution) containing (in mM): KOH 70, L-glutamic acid 50, KCl 55, taurine 20, KH_2_PO_4_ 20, MgCl_2_ 3, EGTA 0.5, HEPES 10 and glucose 20, adjusted to pH 7.2 with KOH, for 5 min. The atrium was then dissected from the heart and placed in a dish. Individual cardiomyocytes were released by mechanical agitation and stored at 4°C in KB solution.

### Electrophysiological measurements

Acetylcholine-activated K^+^ currents (*I_KACh_*) in the whole-cell configuration were recorded using the perforated patch clamp technique [18]. Single atrial cells were placed in a recording chamber attached to an inverted microscope (IMT-2; Olympus, Tokyo) and superfused with normal Tyrode's solution at a rate of 3 ml/min. All experiments were performed at room temperature. Patch pipettes were made from glass capillaries with a diameter of 1.5 mm using a microelectrode puller (Sutter Instruments, P-97) and were filled with solution to a resistance of 2–3 MΩ. The *I_KACh_* was recorded from single isolated myocytes in a perforated patch configuration using nystatin (200 µg/ml; ICN) at room temperature. The composition of the pipette solutions for perforated patches contained (in mM): KCl 140, MgCl_2_ 1, NaH2PO_4_ 0.5, HEPES 10 and EGTA 5, adjusted to pH 7.2 with KOH. *I_KACh_* was activated by extracellular application of acetylcholine (Ach, 100 µM for 2 min), and peak *I_KACh_* was measured as the difference between the peak and the steady-state current at the end of the pulse. After the baseline *I_KACh_* current was measured, varying concentrations of simvastatin or pravastatin were applied for 10 minutes, and a second *I_KACh_* current was recorded. The peak and quasi-steady-state *I_KACh_* recordings (taken before and after 10 minutes of statin treatment, respectively) were then compared. Current signals were recorded using Clampfit 6.0 software (Axon Instruments, Inc., Foster City, CA, USA).

### Materials

Simvastatin, pravastatin, mevalonic acid lactone, and all other chemicals were from Sigma Chemical Co. (St. Louis, MO, USA). Simvastatin was dissolved in dimethyl sulfoxide (DMSO, Amresco), and pravastatin was dissolved in distilled water. Simvastatin was prepared fresh for each experiment from a stock solution (10 mM in DMSO, stored at −20°C) and diluted a final concentration of 10 µM, and added in the bath solution. For each experiment, small aliquots of the HMG-CoA reductase inhibitor stock solutions were added to normal Tyrode's solution. The final concentration of DMSO was 0.1% and had no effecton *I_KACh_* in atrial cardiomyocytes [20].

### Statistical analysis

Statistical analyses were performed using SPSS for Windows, ver. 15.0, (SPSS, Inc., Chicago, IL, USA). Numeric data were expressed as the mean ± SD, and electrophysiological data were presented as the mean ± standard error of the mean (SEM). The statistical differences among the nominal variables of the groups were analyzed using the one-way ANOVA test, and the differences between the subgroups were assessed with the post-hoc Tukey test. A P value of <0.05 was considered statistically significant for all the tests.

## Results

### Effect of simvastatin on *I*_KACh_ in mouse atrial cells

**Figure 1:**
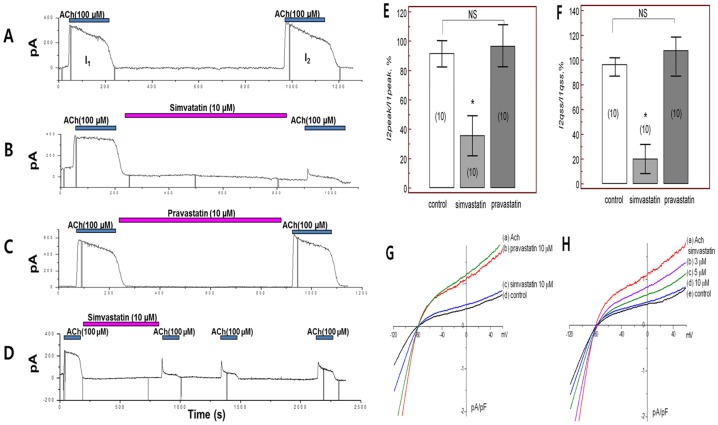
Acetylcholine-activated K^+^ currents (*I_KACh_*) were recorded using a nystatin-perforated whole cell patch clamp technique. A. Acetylcholine (100 µM) was applied to the bath solution, and *I_KACh_* was promptly activated. B. Simvastatin (10 µM) treatment for 10 minutes significantly reduced the peak and quasi-steady-state *I_KACh_* amplitudes. C. Pravastatin (10 µM) treatment for 10 minutes did not change peak or quasi-steady-state *I_KACh_* amplitude. D. After 10 minutes washout of simvastatin, *I_KAch_* was partially recovered. E. Peak amplitude at baseline *I_KACh_* (*I*1, peak) and the second *I_KACh_* peak (*I*2, peak), after statin application. F. Quasi-steady state amplitude of baseline *I_KACh_* (*I*1, qss) and second qss *I_KACh_* (*I*2, qss) after statin application. G. Current–voltage (I–V) curves were plotted. The ramps were applied before (d) and after acetylcholine 100 µM application (a), in the presence of pravastatin 10 µM (b) and simvastatin 10 µM (c). H. The ramps were applied before (e) and after acetylcholine 100 µM application (a), in the presence of simvastatin 3 µM (b), 5 µM (c) and 10 µM (d). NS; no significant change, *; p<0.05 compared to controls.

**Figure 2:**
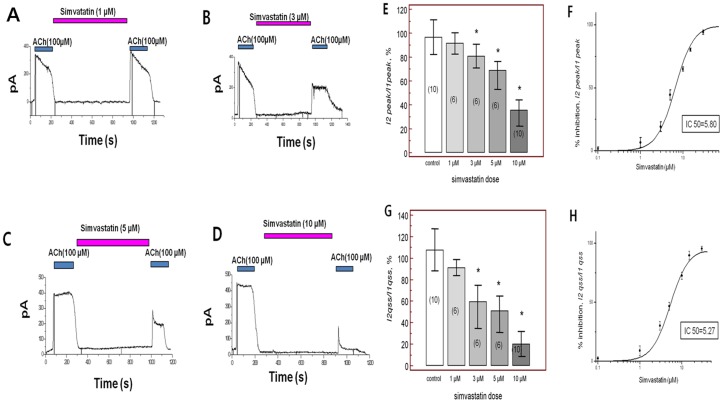
Simvastatin inhibits acetylcholine-activated K^+^ current (*I_KACh_*) in a dose-dependent manner at A. 1 µM, B. 3 µM, C. 5 µM and D. 10 µM. E. Peak amplitude of baseline *I_KACh_* (*I*1, peak) and second *I_KACh_* peak (*I*2, peak) after application of simvastatin. F. Dose response curve for the percent inhibition of peak *I_KACh_* amplitude in the presence of simvastatin. G. Quasi-steady state amplitudes of baseline *I_KACh_* (*I*1, qss) and the second *I_KACh_* (*I*2, qss) after simvastatin. H. Dose response curve for the percent inhibition of quasi-steady state *I_KACh_* amplitude in the presence of simvastatin. NS; no significant change, *; p<0.05 compared to controls.

**Figure 3:**
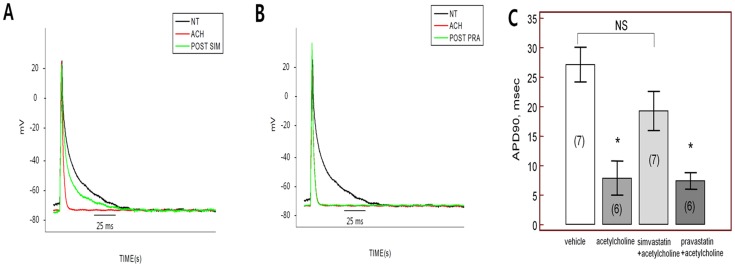
Change of action potential duration (APD) after acetylcholine application with or without statins. A. Acetylcholine significantly shortened APD at 90% repolarization, while simvastatin restored APD to vehicle level (NT, normal tyrode). B. Pravastatin did not restore the shortened APD induced by acetylcholine. C. Comparison of APD at 90% repolarization after acetylcholine application with simvastatin or pravastatin. NS; no significant change, *; p<0.05 compared to controls.

Application of acetylcholine (100 µM) to the bath solution promptly activated *I*_KACh_ in mouse atrial myocytes (Fig. 1*A*). Re-application of acetylcholine after washout for >10 min induced *I_KACh_* to a similar amplitude (Fig. 1*A*), indicating reproducibility of *I_KACh_* during the investigation period. We next examined the effects of simvastatin on *I_KACh_*. After baseline *I_KACh_* measurement (I1), simvastatin (10 µM) was applied for 10 minutes, and *I_KACh_* in the presence of simvastatin (I2) was compared to baseline *I_KACh_* (I1). As shown in Fig. 1*B*, treatment with simvastatin for 10 min significantly reduced peak *I_KACh_* current. After 10 minutes washout of simvastatin, *I_KAch_* was partially recovered 76.4±11.3% of baseline current (Fig. 1*D*). On average, peak *I*2 (*I*2, peak) was 35.5±13.6% of *I*1 (*I*1, peak), while the quasi-steady-state amplitude of *I*2 (*I*2, qss) was 19.9±11.8% of *I*1 (*I*1, qss) (p<0.001 for the I2 peak and p<0.001 for the I2 qss (each n = 10, Figs. 1*E–F*). Current–voltage (I–V) curves were obtained from the current response induced by voltage ramps between −120 and +60 mV from the holding potential of −40 mV. Corresponding I–V curves were plotted in Fig. 1 G, H and I–V relationships demonstrated that simvastatin inhibited the net *I_KACh_* over the whole tested voltage range. In addition, simvastatin inhibited *I_KACh_* in a dose-dependent manner between 1 and 10 µM (1 µM, n = 6; 91.5±9.0%, 3 µM, n = 6; 80.8±9.9%, 5 µM, n = 6; 68.7±15.7%, 10 µM, n = 10; 35.5±13.6%, p<0.001, Fig. 2), which was also shown in I-V relationships (Fig. 1H). When we tested the effect of simvastatin on the *I_KAch_* without acetylcholine administration, simvastatin had no influence on the *I_KAch_* over the whole tested voltage range (n = 3, Fig. S1A). When we test a time dependent effect for achieving steady-state block of *I_KACh_*, there were no significant differences in achieving steady-state block of *I_KAch_* among 5 min, 10 min, and 15 min after simvastatin application (each n = 5, p = NS, Fig. S2). The percent inhibition in the presence of simvastatin was calculated with respect to the amplitude of peak (*Ipeak*) and quasi-steady-state (*Iqss*) in the presence of simvastatin and plotted in Fig. 2F and 2H. The data were fitted with the Hill equation, showing that the concentration for half-maximal inhibition (IC50) was 5.80 µM for the *Ipeak* and 5.27 µM for the *Iqss* (n = 3 in every points, total n = 21). Importantly, acetylcholine significantly shorted APD at 90% repolarization (APD_90_, from 27.3±2.2 ms to 7.2±1.6 ms, p<0.01), while treatment with simvastatin recovered levels to those of vehicle treatment (vehicle, n = 7; 27.3±2.2 ms, simvastatin, n = 7; 19.3±3.3 ms, p = 0.34, Figs. 3*A, C*). When we tested the effect of simvastatin on the APD_90_ without acetylcholine, simvastatin had no influence on the APD_90_ (n = 3, Fig. S1B).

### Effect of pravastatin on *I_KACh_* in mouse atrial cells

We next investigated the effects of pravastatin on *I_KACh_* using the same experimental protocol. Addition of pravastatin in a bath solution for 10 minutes did not significantly alter peak amplitude or quasi-steady-state of the currents compared to controls (n = 10, p = 0.48 for peak *I_KAch_* and n = 10, p = 0.19 for qss *I_KAch_*, Figs. 1*C, E, F*) and did not restore the acetylcholine-induced shortening of APD (acetylcholine, n = 6; 8.5±3.7 ms, pravastatin, n = 6; 9.1±4.3 ms, p = 0.85, Figs. 3*B, C*).

### Mechanism of simvastatin-induced *I_KACh_* inhibition in mouse atrial cells

**Figure 4:**
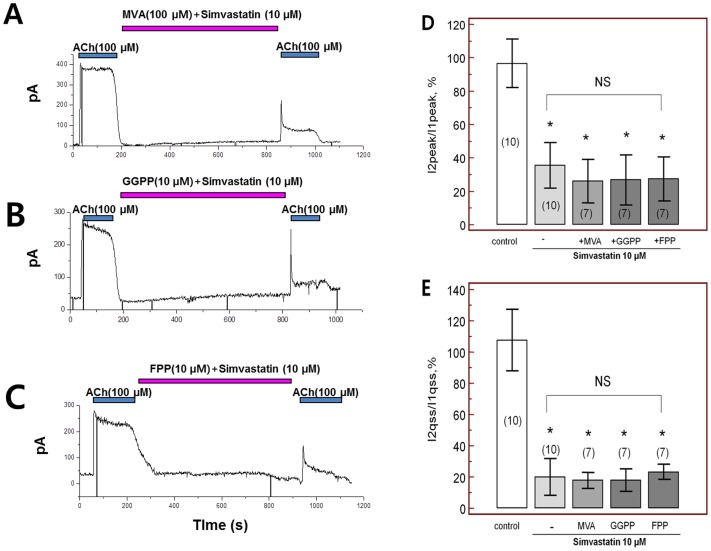
Acetylcholine-activated K^+^ current (*I_KACh_*) after simvastatin in the presence of substrates and intermediary metabolites in cholesterol synthesis; A. mevalonate (MVA), B. geranylgeranyl pyrophosphate (GGPP), and C. farnesyl pyrophosphate (FPP). D. Peak amplitudes at baseline *I_KACh_* (*I*1, peak) and the second *I_KACh_* peak (*I*2, peak) after simvastatin with various cholesterol biosynthetic intermediates. E. Quasi-steady state amplitude of baseline *I_KACh_* (*I*1, qss) and second *I_KACh_* (*I*2, qss) after simvastatin with various cholesterol biosynthetic intermediates. NS; no significant change, *; p<0.05 compared to controls.

**Figure 5:**
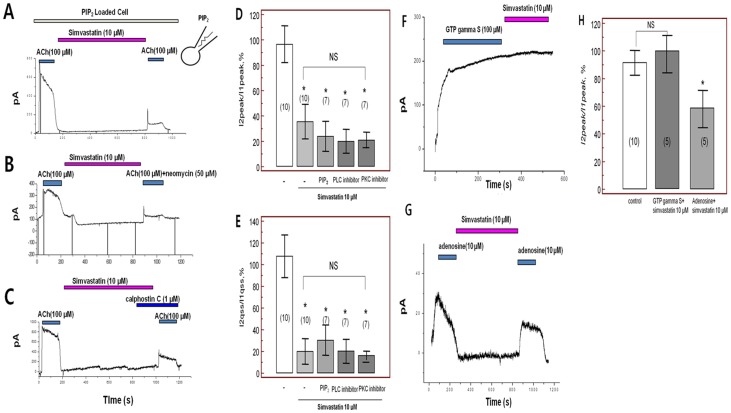
Acetylcholine-activated K^+^ current (*I_KACh_*) after simvastatin with A. phosphatidylinositol 4,5-bisphosphate (PIP_2_), B. phospholipase C inhibitor (PLC inhibitor, neomycin 50 µM), and C. protein kinase C inhibitor (PKC inhibitor, calphostin C 1 µM). D. Peak amplitudes of baseline *I_KACh_* (*I*1, peak) and the second *I_KACh_* (*I*2, peak) after treatment with PIP_2_, PLC inhibitor, and PKC inhibitor. E. Quasi-steady state amplitudes of baseline *I_KACh_* (qss *I*1) and second *I_KACh_* (qss *I*2) after treatment with PIP_2_, PLC inhibitor, and PKC inhibitor. F. Simvastatin did not inhibit the activated *I_KACh_* by intracellular loading of GTP gamma S (100 µM/L). G. Simvastatin inhibit activated *I_KACh_* by adenosine. H. Peak amplitudes of baseline *I_KACh_* (qss *I*1) and second *I_KACh_* (qss *I*2) activated by GTP gamma S and adenosine after treatment with simvastatin. NS; no significant change, *; p<0.05 compared to controls.

To investigate the association between simvastatin-induced *I_KACh_* inhibition and inhibition of cholesterol synthesis, substrates for cholesterol synthesis consisting of mevalonate (MVA, Fig. *4A*), geranylgeranyl pyrophosphate (GGPP, Fig. *4B*), or farnesyl pyrophosphate (FPP, Fig. *4C*) were added with simvastatin in the bath solution. However, the reductions in peak amplitude and quasi-steady-state current of *I_KACh_* by simvastatin were not prevented by supplementation with any of these substrates (p = 0.28, p = 0.37 and p = 0.41 for MVA, GGPP and FPP, respectively, each n = 7, Figs. 4*D,E*). Moreover, to investigate if the modulation of simvastatin-induced *I_KACh_* inhibition may happen through the phospholipase C (PLC), protein kinase C (PKC) pathway or depletion of phosphatidylinositol 4,5-bisphosphate (PIP_2_) [19], [20], PLC inhibitor, PKC inhibitor, and PIP2 were tested. Loading the patch pipette with PIP_2_ via whole cell ruptured patch clamp did not alter simvastatin-mediated inhibition of *I_KACh_* (Fig. *5A*), implying that simvastatin did not limit the availability of these agents. Similarly, application of the PLC inhibitor neomycin (50 µM, Fig. *5B*) or the PKC inhibitor calphostin C extracellular solution (1 µM, Fig. *5C*) failed to alter simvastatin-inhibition of *I_KACh_* (each n = 7, Figs. *5D,E*). When we activate *I_KACh_* by intracellular loading of GTP gamma S (100 µM/L) via whole cell patch, simvastatin did not inhibit *I_KACh_* (n = 5, Figs. *5F,H*). However, when we activate *I_KACh_* by extracellular application of adenosine, simvastatin also inhibit adenosine activated *I_KACh_* (n = 5, Figs. *5G,H*), which suggest that simvastatin influence on the adenosine binding site as well as acetylcholine binding sites. This result suggests that acute administration of simvastatin may inhibit the *I_KACh_* by blockade of acetylcholine binding site.

## Discussion

The results of this study indicated that lipophilic simvastatin but not hydrophilic pravastatin suppressed *I_KACh_* in mouse atrial myocytes. These effects were not dependent on cholesterol biosynthesis or PIP_2_ pathway, suggesting the involvement of direct inhibition of *I_KACh_*. In addition, simvastatin significantly attenuated acetylcholine-induced APD shortening. Importantly, these results provided the first direct evidence that the lipophilic HMG CoA reductase inhibitor simvastatin facilitates its potent anti-arrhythmic effect by inhibiting *I_KAch_* and suppressing electrical remodeling in mammalian atrial myocytes.

### Effects of statins on *I*_KACh_ in mouse atrial cells

Statins exert pleiotropic effects in part by reducing the availability of intermediary metabolites in cholesterol synthesis (isoprenoids), which in turn mediate regulatory signaling through activation of guanosine nucleotide-binding proteins (G-proteins). Through G-protein inhibition, treatment with statins may induce rapid and significant improvement in endothelial function [21], in part by reversing the suppression of endothelial nitric oxide synthase [23] associated with hypercholesterolemia [21], [22].

The effectiveness of statins in both primary and secondary prevention of AF implies that multiple mechanisms may be involved in their anti-arrhythmic activity. The capacity of statins to reduce inflammation, thereby reducing the risk of AF [24], may reflect the pleiotropic properties of these drugs, in part because they are independent of the lipid-lowering effects. Although a direct causative relationship between inflammation and AF has not been established [25], inflammation may induce autonomic remodeling, providing a substrate for initiation and maintenance of AF [26]. In addition to indirect anti-arrhythmic effects, statins may also act directly by modulating fatty acid composition and physiochemical properties of cell membranes, resulting in alterations of the properties of transmembrane ion channels [7], [27]. The established role of atrial tachycardia–induced electrical remodeling in AF [28], [29] implies that changes in ion channel function (“ionic remodeling”) are involved in this pathophysiological process [28]–[30], and thus statins may in turn influence ion channel activities. Seto et al. [31] reported that simvastatin inhibits Ca^2+^-activated K^+^ channels in arterial smooth muscle cells, while Bergdahl *et al.* [32] showed that lovastatin inhibits L-type Ca^2+^ currents in rat basilar artery smooth muscle cells. Atorvastatin and simvastatin produce a concentration-dependent blockade of hKv1.5 channels *in vitro* [33]. In addition, simvastatin attenuates cerebrovascular remodeling in the hypertensive rat through inhibition of vascular smooth muscle cell proliferation by suppression of volume-regulated chloride channels [13].

Upregulation of inwardly rectifying potassium channels is an important contribution to the electrical remodeling underlying AF. Accordingly, inhibition of these currents may be a potential anti-arrhythmic target devoid of ventricular side effects [34]. We previously demonstrated that the constitutively active *I_KACh_* substantially contributes to the repolarization phase of atrial action potential in AF. Further, as a potential ionic determinant of AF, *I_KACh_* represents a plausible target for therapy [35]–[37]. The results of the present study confirmed our hypothesis that treatment with the lipophilic statin simvastatin but not with hydrophilic pravastatin attenuates *I_KACh_* as a component of the anti-arrhythmic effect of statins.

### Contrasting effects of simvastatin and pravastatin on *I*_KACh_ in mouse atrial cells

Clinical trials of statins in the prevention of AF recurrence have reported mixed results. Although atorvastatin and simvastatin reduced AF recurrence after electrical cardioversion (EC) [6], use of pravastatin before EC did not decrease AF recurrence [9] and rosuvastatin did not affect clinical outcome and AF occurrence [10], [11]. Lipophilic statins improve cardiac sympathetic activity by reducing oxidative stress [38], [39], and an active metabolite of atorvastatin displays stronger antioxidant activity than rosuvastatin [40]. Simvastatin but not pravastatin significantly reduces angiotensin II-induced calcium mobilization [41], and simvastatin may exert direct anti-arrhythmic effect by suppressing events that trigger AF [42]. Accordingly, our results indicate that the inhibition of *I_KACh_* may represent another important anti-arrhythmic mechanism of simvastatin. This inhibitory action on the *I_KACh_* current was not reversed by addition of mevalonate (MVA), GGPP, or FPP, implying that simvastatin may suppress *I_KACh_* independently from signaling proteins activated by isoprenylation. Moreover, PLC/PKC inhibition and PIP_2_ supplementation did not change simvastatin induced *I_KACh_* inhibition, implicating that statin-induced *I_KACh_* inhibition is independent of PKC pathway. Interestingly, simvastatin also inhibit adenosine activated *I_KACh_*, which suggest that simvastatin influence on the adenosine binding site as well as acetylcholine binding sites. However, intracellular application of gamma GTP 100 µM/L induced *I_KACh_* activation was not suppressed by simvastatin, which possibly suggest that simvastatin induced *I_KACh_* inhibition may be done by interference of acetylcholine ligand binding pocket. We observed the inhibition of *I_KACh_* as soon as 10±20 sec after administration of simvastatin, which suggested that inhibition of *I_KACh_* does not involve metabolism of the drug but occurs through direct interaction of the drug with K^+^ channels within the membrane. The highly lipophilic simvastatin has a strong affinity for the cell membrane [13] and, consequently, it may has easy access to the intracellular space; this may explain the ability of simvastatin to effectively inhibit *I_KACh_* in atrial myocytes. In contrast, hydrophilic pravastatin has limited access to the plasma membrane and intracellular space [13], which may explain the absence of immediate effect on *I_KACh_*. In accordance with our results, Matsuda et al. [43] showed that the inhibitory effect of simvastatin on catecholamine secretion induced by acetylcholine does not involve its inhibition of mevalonate-derived isoprenoid synthesis, and that pravastatin does not inhibit acetylcholine-induced catecholamine secretion in cultured adrenal medullary cells. Pravastatin significantly increases parasympathetic modulation of heart rate by stimulation of Gα (i2) expression [44] and protects against ventricular arrhythmias [45], while parasympathetic stimulation is known to promote AF through shortening of atrial refractory periods.

It should be noted that we studied acute exposure rather than chronic treatment, which should be taken into consideration in addition to the extreme caution that must be taken when extrapolating results from mouse atrial cardiomyocytes to human disease. Moreover, we did not manipulate membrane cholesterol and did not study the gating kinetics, and these will be interesting future research themes. In conclusion, we found that the lipophilic statin simvastatin suppressed acetylcholine-activated *I_KACh_*, while the hydrophilic statin pravastatin did not. These results provide important background information for using lipophilic statins in the clinical treatment of AF.

## Supporting Information

Figure S1 A. Simvastatin had no influence on the *I_KAch_* over the whole tested voltage range without acetylcholine. B. Simvastatin had no influence on the APD_90_ without acetylcholine.
(TIF)

Figure S2 **To investigate a time dependent effect, steady-state block of** ***I_KAch_*** **were achieved at the 5 minute, 10 minute, and 15 minutes after simvastatin application.** There were no significant differences in achieving “steady-state” block of *I_KAch_* among 5 min, 10 min, and 15 min (each n = 5, total n = 15, p = NS). NS = no significant change.
(TIF)
