# Supplementary figures and images for: Attenuation of Acetylcholine Activated Potassium Current (IKACh) by Simvastatin, Not Pravastatin in Mouse Atrial Cardiomyocyte: Possible Atrial Fibrillation Preventing Effects of Statin
Source: PLoS One. 2014 Oct 16;9(10):e106570. doi: 10.1371/journal.pone.0106570 (PMC4199526; doi:10.1371/journal.pone.0106570)

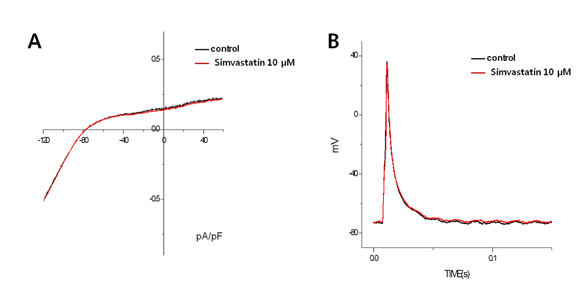

Supplement: Figure S1 — A. Simvastatin had no influence on the IKAch over the whole tested voltage range without acetylcholine. B. Simvastatin had no influence on the APD90 without acetylcholine. (TIF) [file pone.0106570.s001.tif]

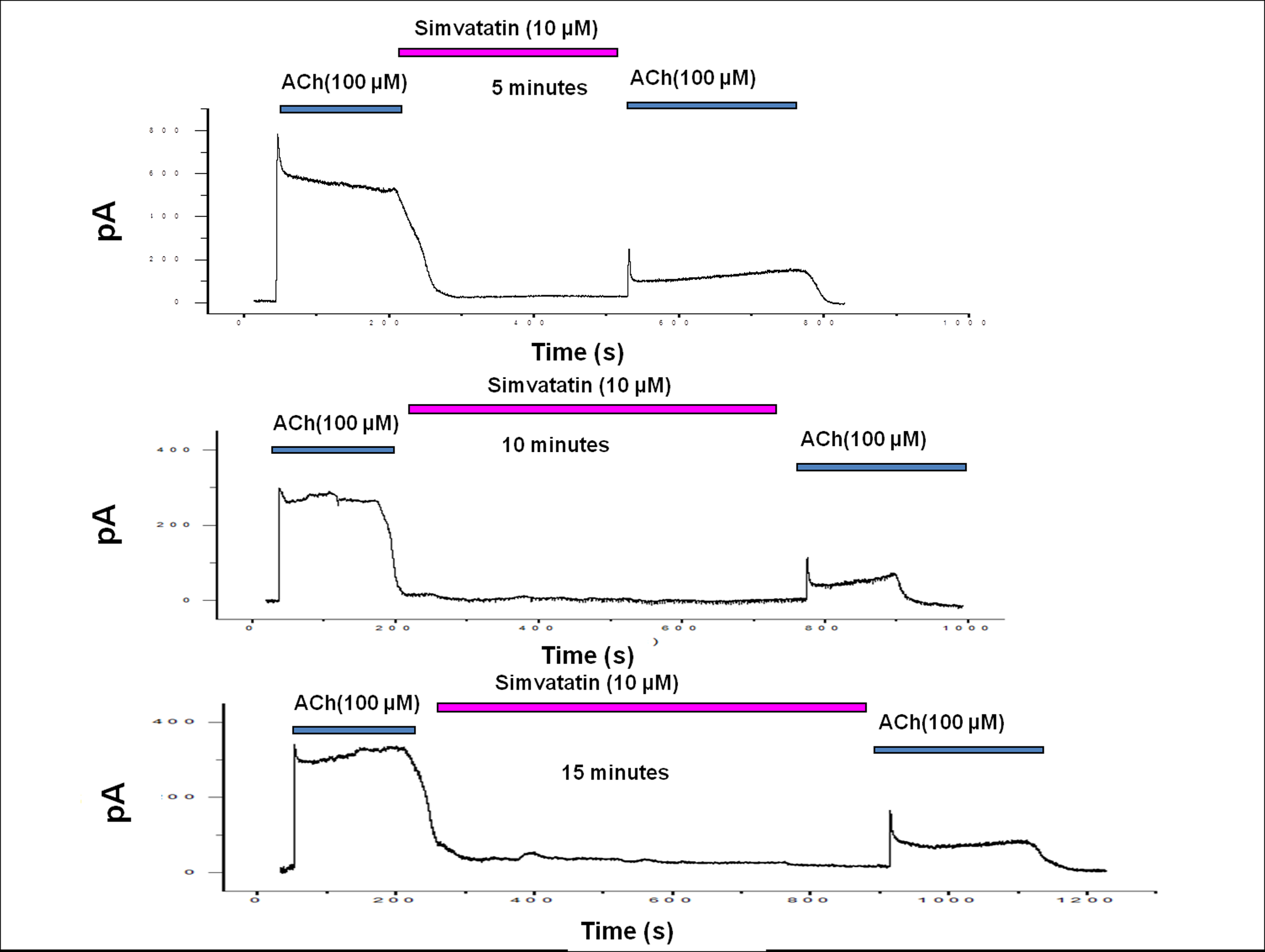

Supplement: Figure S2 — To investigate a time dependent effect, steady-state block of IKAch were achieved at the 5 minute, 10 minute, and 15 minutes after simvastatin application. There were no significant differences in achieving “steady-state” block of IKAch among 5 min, 10 min, and 15 min (each n = 5, total n = 15, p = NS). NS = no significant change. (TIF) [file pone.0106570.s002.tif]
